# Brain Activity during Lower-Limb Movement with Manual Facilitation: An fMRI Study

**DOI:** 10.1155/2015/701452

**Published:** 2015-02-02

**Authors:** Patrícia Maria Duarte de Almeida, Ana Isabel Correia Matos de Ferreira Vieira, Nádia Isabel Silva Canário, Miguel Castelo-Branco, Alexandre Lemos de Castro Caldas

**Affiliations:** ^1^Alcoitão School of Health Sciences, Rua Conde Barão, Alcoitão, 2649-506 Alcabideche, Portugal; ^2^Institute of Health Sciences, Catholic University of Portugal, Palma de Cima, 1649-023 Lisbon, Portugal; ^3^Visual Neuroscience Laboratory, Institute for Biomedical Imaging in Life Sciences (IBILI), ICNAS, Faculty of Medicine, University of Coimbra, Azinhaga de Santa Comba, 3000-548 Coimbra, Portugal

## Abstract

Brain activity knowledge of healthy subjects is an important reference in the context of motor control and reeducation. While the normal brain behavior for upper-limb motor control has been widely explored, the same is not true for lower-limb control. Also the effects that different stimuli can evoke on movement and respective brain activity are important in the context of motor potentialization and reeducation. For a better understanding of these processes, a functional magnetic resonance imaging (fMRI) was used to collect data of 10 healthy subjects performing lower-limb multijoint functional movement under three stimuli: verbal stimulus, manual facilitation, and verbal + manual facilitation. Results showed that, with verbal stimulus, both lower limbs elicit bilateral cortical brain activation; with manual facilitation, only the left lower limb (LLL) elicits bilateral activation while the right lower limb (RLL) elicits contralateral activation; verbal + manual facilitation elicits bilateral activation for the LLL and contralateral activation for the RLL. Manual facilitation also elicits subcortical activation in white matter, the thalamus, pons, and cerebellum. Deactivations were also found for lower-limb movement. Manual facilitation is stimulus capable of generating brain activity in healthy subjects. Stimuli need to be specific for bilateral activation and regarding which brain areas we aim to activate.

## 1. Introduction

The knowledge of normal brain activity during several tasks gives insight for both normal and abnormal behavior [1]. Brain activity knowledge of healthy subjects is an important reference in the context of motor control. This understanding of mechanisms underlying motor control and relearning is the basis for neurosciences development of frameworks for motor performance potentialization or reeducation. In the context of neurorehabilitation, this is shown in the recovery of disturbances which tend to present similar brain networks to those of healthy subjects [2–4] as the result of neuroplasticity [5].

Brain behavior is a complex task, being related with several aspects like somatotopic identification, activations and deactivations [6], sequences and differentiations of activations, interconnectivity, metabolic changes, and synaptic transmissions, among others.

While the normal brain behavior for upper-limb motor control has been widely explored, the same is not true for lower-limb control. It is however known that, in addition to motor and premotor areas, other areas such as somatosensory and limbic areas and basal nuclei and cerebellum structures are involved in the process of motor control [7, 8] of healthy subjects. Specifically, homunculus representations of the lower limb on motor and somatosensory and cerebellum areas are activated [9]. However, most of the studies refer to single-joint movements, not reflecting the complexity of functional movements. Thus, the identification of somatotopic maps of brain activity during complex movements of lower limbs on healthy subjects is still needed for the understanding of mechanisms underlying motor control of lower limb.

Considering the need for synaptic selection of activations and inhibitions, for shaping patterns of activity in networks underlying complex skills, both activations and deactivations are important in brain activity analysis [6]. Deactivations are a controversial issue in brain imaging, as the interpretations are not yet clear or well established [6]. They appear to be associated with decreases in blood oxygen levels dependent signal (BOLD), usually associated with the inhibition of areas not involved in the specific task in order to facilitate task-relevant processing [2].

As movement can be triggered by different stimuli like cognition, motivation, verbal orders, vision, external manual guidance, environment, and task demands, other areas than motor-related areas are expected to be involved in the process of neural connections. Also the experience-dependent process of the dominant or nondominant limb [10] will influence the localization, the intensity, and the pattern of brain activity.

On the perspective of movement potentialization or reeducation, the understanding of the impact of the different stimuli on motor-related areas is relevant for a selection of the closest-to-normal autonomous movements and the scientific base for professions like physiotherapy.

The latest research studies already show some evidence for brain activation through several physiotherapeutic approaches in both healthy subjects and neurological patients [11–15]. However, none of the studies focused on external manual guidance or “manual facilitation,” the most frequently used stimulus considered as the conventional physiotherapy treatment [16]. The underlying neurophysiological processes that are elicited by motor-related sensory stimuli during manual facilitation have not been previously investigated. Its empirical use relies on the assumptions that activation of tactile and proprioceptive receptors will activate the somatosensory areas (S1 and S2) creating a body map at the homunculus and insula region [17]. As the insula is also responsible for motor functions, by the activation of the anterior cingulate [18], it is expected that the manual stimulation has effects on motor and somatosensory activation.

With regard to these considerations concerning brain activity, physiotherapeutic stimuli, and the complex movements of lower limbs, the goal of this whole-brain functional MRI study is to analyse the somatotopic map of brain activity for lower limbs during multijoint functional movement (simultaneous movement of the hip, knee, and ankle) and to investigate the effects of the manual facilitation of lower-limb functional movements on brain activity in healthy subjects.

To that end, we analysed brain activity through three different stimuli for movement performance: (a) verbal stimulus; (b) manual stimulus (physiotherapeutic manual facilitation); and (c) verbal + manual stimulus.

In contrast with other studies, we analysed multijoint movement of the lower limb during complex functional tasks and not single-joint movements, the brain activity during the performance of manual facilitation of movement using a specific physiotherapeutic approach and not after a period of intervention, and the white matter activity and attempted to analyse deactivations.

## 2. Methods

### 2.1. Participants

A sample of 10 healthy subjects (5 males/5 females; mean age of 60.6 ± 9.1 years), right-handedness and footedness assessed by the Portuguese-language translation of the Waterloo Handedness Questionnaire-Revised (WHQ-R) and Waterloo Footedness Questionnaire-Revised (WFQ-R) [19], participated in this study. They presented no relevant medical history and no indicators of anxiety on the State-Trait Anxiety Inventory (STAI) [20] scale or mental disorders on the Saint Louis University Mental Status (SLUMS) [21] scale or negative social touch reaction according to the Social Touch Questionnaire (STQ) [22] (Table 1). The experimental procedures were approved by the Ethics Committee of Health Sciences Institute at the Portuguese Catholic University and all participants gave their informed consent in accordance with the Declaration of Helsinki prior to their participation.

### 2.2. Procedures for Brain Activity Acquisition

#### 2.2.1. Functional Magnetic Resonance Imaging Scanning

Data acquisition was performed on a 3 Tesla scan Siemens Magnetom Trio at the Portuguese Brain Imaging Network. A whole-brain approach, starting with one 3D anatomical MPRAGE sequence T1-weighted, 1 × 1 × 1 voxel size, repetition time (TR): 2,530 ms, echo time (TE): 3.42 ms, field of view (FOV): 256 × 256 mm, and a matrix size of 256 × 256. The anatomical sequence comprised 176 slices. Functional MRI experiment was acquired in 2 functional runs: RUN 1, right lower limb (RLL), and RUN 2, left lower limb (LLL), in the same session, sensitive to BOLD signal sequences, a TR: 2500 ms, TE: 30 ms, voxel size 3 × 3 × 3 mm, FOV: 256 × 256, and a matrix size of 86 × 86. For each run, 45 slices were acquired with 200 volumes.

#### 2.2.2. Experimental Paradigms/Motor Testing

All participants underwent a single session comprising one structural scan and one functional scan with two runs. Both runs consisted of 3 stimulation blocks and 1 fixation block (Table 2). The stimulation blocks aimed to induce the movement of lower limbs in a pattern of hip flexion, knee flexion, and dorsiflexion, requiring multijoint movement and a stabilization of the contralateral side, with the following stimuli:

- *Block 1—verbal stimulus*, “bring your leg up to the table,” recorded on a sound recorder with a female voice and translated into audio windows media format and listened to by the subjects—to be used as a trigger for autonomous movement performance and consequently create an expected somatotopic map of activation closed to the voluntary autonomous movement;
- *Block 2—physiotherapeutic manual facilitation stimulus* based on Bobath concept key points [23], performed by a specialized physiotherapist, encouraging the movement of the leg up to the table, with one hand on the dosal face of the foot, stimulating manually the movement of dorsiflexion, and another hand on the external superior extremity of lower leg stimulating knee elevation, leading to hip flexion—to verify the effects of manual stimulus;
- *Block 3—mixed stimuli* including both verbal and physiotherapeutic manual facilitation—to verify if any stimulus is predominant over the other.

Each stimulation block included 5 trials each lasting 7 seconds, totalling 35 seconds per stimulation block with a total of 105 seconds of stimulation per run. Resting periods of 15 seconds were used after each trial for the repositioning of the LL. The fixation block lasted 30 seconds, being applied before the first stimulation trial and after the last stimulation trial. The fixation block served baseline purposes and the participants were asked to rest and make no intentional movement. The sum of this time came to 322 seconds. The overall functional acquisition lasted 990 seconds for each subject. The functional acquisition always started with the RLL and the sequence of the following stimulation blocks was the same to all subjects and was previously randomised on Matlab R 2013a, for preparation of the physiotherapist performing the stimulus but no anticipation of the subject. Three different image codes were displayed on a computer screen for each block only for the physiotherapist. This procedure allowed the physiotherapist to identify the blocks when his participation was needed and showed the necessary duration.

### 2.3. Image Processing and Data Analysis

Functional imaging analysis was carried out using BrainVoyager QX version 2.3 software (Brain Innovation B.V., Netherlands; http://www.brainvoyager.com/). Anatomical images were reoriented into a space where the anterior and the posterior commissure lie on the same plane (AC-PC) and then transformed to the Talairach reference system. Functional images were intensity-adjusted and all slice scans were time- and 3D-motion-corrected, temporal-filtered, and subsequently coregistered to the structural image. The first three functional volumes were discarded in order to attain signal equilibrium.

The effects of stimulation blocks versus baseline were determined by performing, for each functional run, a one-way repeated ANOVA measure for the identification of significant clusters for each contrast. Due to the presence of substantial head movements caused by the design of the experience itself, it was deemed necessary to include 6 motion confound predictors (*x*, *y*, *z*, rotation, and translation) into the whole-brain Random Effects-General Linear Model Analysis (RFX-GLM). This allowed for the possibility for generalization to the population [24]. In addition, a whole-brain mask was included in order to eliminate voxels located outside of the boundaries of the brain. We considered the presence of significant clusters at the 0.05 threshold, corrected for multiple comparisons using a cluster threshold estimator (based on Monte Carlo simulations (1,000 interactions)). The cluster-size thresholding allowed us to define multisubject volumes of interest (VOIs), according to the clusters' center of mass (CoM), and measure its activation volume. We also examined the surrounding areas that were included in the identified clusters using the Brain Voyager Brain Tutor atlas. These areas were properly identified according to the location of their center of mass and peak voxel, but no activation volume was recorded due to the intrinsic limitations of using a brain atlas in order to segment these areas. The VOIs were obtained using particular contrasts. The contrast of* verbal stimulus* with the baseline would be used to provide a somatotopic map of reference for the lower-limb multijoint movement of healthy subjects; the contrast of the* manual stimulus* with the baseline would be used to verify the effects of manual facilitation on brain activity; and the contrast of the manual + verbal stimulus with the baseline would be used to identify if there is any advantage in giving simultaneous stimuli. Specific predictors from the stimulation blocks were compared:* verbal stimulus* >* manual stimulus; manual stimulus* >* verbal stimulus*.

## 3. Results

### 3.1. Brain Activity during Verbal Stimulus for the Multijoint Movement of Lower Limbs

For both lower limbs, verbal stimulus for movement elicits a statistically significant (RFX, *P* = 0.05, corrected) bilateral midline cortical brain activation in the M1, S1, S2, and cingulate cortex.

**Figure 1:**
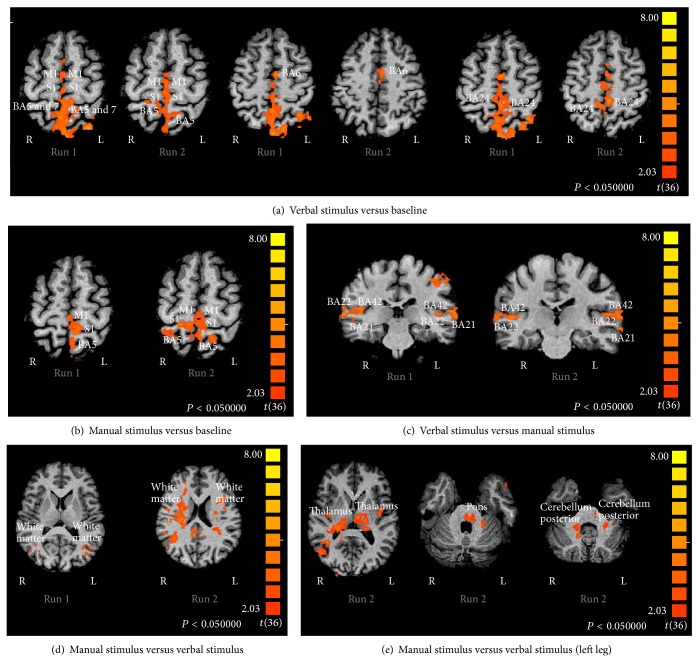
Statistical maps of activation for lower-limb movement. BA: Brodmann area; R: right hemisphere; L: left hemisphere; Run 1: right leg; Run 2: left leg.

For the RLL, the cluster with the greatest volume of activation has both its Center of Mass and its Peak Voxel level at S2-BA7 (number of voxels = 16,655; *t*(0.36) = 6.58; *P* < 0.00 for the right hemisphere and number of voxels = 2080; *t*(0,36) = 5.60; *P* < 0.00 for the left hemisphere) and includes primary somatosensory (BA1, 2, and 3) and motor areas (BA4) and cingulate cortex areas (BA24, 30, 31, and 32) (see Figure 1(a), Table 3(a), and Appendix 1 in Supplementary Material available online at http://dx.doi.org/10.1155/2015/701452).

For the LLL (see Figure 1(a), Table 3, and Appendix 1), the cluster with the greatest volume has both its Center of Mass and its Peak Voxel level at M1-BA4 (number of voxels = 7,153; *t*(0.36) = 5.02; *P* < 0.00 for the right and left hemispheres) and includes the same areas as the RLL.

We also found activation in SMA-BA6, in the left hemisphere for both lower-limb stimulations included in the clusters presented above.

In the areas BA1, 2, 3, 4, 5, and 7, activation is located in the lower-limb representation (homunculus).

Deactivation is found in the interhemispheric connectivity region and occipital area (see Table 3(b)).

Compared with* manual stimulus, verbal stimulus* elicits activity in language (BA21 and 22) and auditory (BA42) areas bilaterally for both lower limbs (see Figure 1(c), Table 3(a), and Appendix 1). Deactivations are found for the RLL, in ipsilateral auditory, visual, language, memory, and subcortical areas and for the LLL in the cerebellum (see Table 3(b)).

### 3.2. Brain Activity during Manual Facilitation of Lower-Limb Multijoint Movement

For the RLL, manual facilitation of movement elicits a statistically significant (RFX, *P* = 0.05, corrected) level of contralateral cortical brain activation. The cluster with the greatest volume of activation has both its Center of Mass and its Peak Voxel level at BA1 (number of voxels = 4,784; *t*(0.36) = 4.98; *P* < 0.00) and includes the primary somatosensory areas (BA2 and 3), the secondary somatosensory area homunculus (BA5 and 7), and the motor area (BA4) (see Figure 1(b), Table 3(a), and Appendix 1). In areas BA1, 2, 3, 4, 5, and 7, activations are located in the lower-limb representation (homunculus).

For the LLL, manual facilitation of movement elicits a statistically significant (RFX, *P* = 0.05, corrected) bilateral cortical brain activation. The cluster with the greatest volume of activation has both its Center of Mass and its Peak Voxel level at BA5 (number of voxels = 11,004;  *t*(0.36) = 5.29; *P* < 0.00) and includes the primary somatosensory areas (BA1, 2, and 3), the secondary somatosensory areas (BA5 and 7), and the motor area (BA4) (see Figure 1(b), Table 3(a), and Appendix 1). Deactivations are found in auditory and linguistic areas as well as in ipsilateral motor, executive, memory, and cognitive areas and upper-limb representation is found in the cerebellum (see Table 3(b)).

Compared with* verbal stimulus*,* manual stimulus* elicits bilateral activity in the white matter of somatosensory areas (both the Center of Mass and the Peak Voxel), with a volume of 42,725 voxels (*t*(0.36) = 5.44; *P* < 0.00) (see Figure 1(d), Table 3(a), and Appendix 1).

For the same contrast, when the LLL is stimulated, bilateral activation is found in SMA-BA6, BA24, and cerebellum (lobes XI and VIIIb). Ipsilateral activation of subcortical areas (thalamus, pons, and amygdala) is also observed (see Figure 1(e), Table 3(a), and Appendix 1). In this comparison, deactivations are found in linguistic and auditory areas for both lower limbs (see Table 3(b)).

### 3.3. Brain Activity during Manual + Verbal Stimuli for the Multijoint Movement of Lower Limbs

The clusters with the greatest volume of activation are related to auditory areas bilaterally.

For the RLL, the Center of Mass is at BA42 (number of voxels = 5,054 in the right hemisphere and 4,276 in the left hemisphere) with the Peak Voxel at BA22 (*t*(0.36) = 5.50, *P* < 0.00, for the right hemisphere and *t*(0.36) = 6.01, *P* < 0.00, for the left hemisphere) (Table 3(a) and Appendix 1).

For the LLL, the Center of Mass is at BA42 (number of voxels = 9,426) with the Peak Voxel level at BA52 (*t*(0.36) = 6.61; *P* < 0.00) in the right hemisphere and at BA22 (number of voxels = 4,829) with the Peak Voxel level at BA22 (*t*(0.36) = 5.59; *P* < 0.00) in the left hemisphere (Table 3(a) and Appendix 1).

For the LLL, bilateral activation was also found in the primary somatosensory areas (BA1, 2, and 3), secondary somatosensory area homunculus (BA5 and 7), ventral cingulate cortex (BA24), and motor area (BA4). Contralateral activation was found in the same areas for RLL.

For the RLL, deactivation of cerebellum and subcortical areas was found. For the LLL, deactivations were found on motor planning and somatosensory areas.

## 4. Discussion

Coherently, the manual stimulus of RLL elicits contralateral cortical activation, requiring less connectivity, probably related with automated mechanisms for the dominant limb and hemisphere.

Despite the analysis of white matter activation being unusual in fMRI studies, we valued it as it represents the cluster with the highest volume of activation. Its localization in the frontal and parietal lobes is coherent with the connectivity of premotor, motor, and somatosensory areas, showing greater activity for the manual stimuli and consequently descending motor information.

The activation of subcortical areas for the LLL manual stimuli may be related with the phenomenon that the nonverbal stimuli do not generate motivation and free-will, requiring more proprioceptive feedback and spatial references for adequate motor programming. This idea is emphasized by the results of the mixed stimulus, where the verbal stimuli do not appear to elicit the subcortical areas and maintain the same activated areas as in the verbal stimulus alone.

The activation of auditory and visual areas must be related with the processing of the sound information and the interpretation of the words related with movement and body segments, generating a more cognitive process for movement performance.

Despite the lack of consensus regarding their interpretation, the deactivations found are coherent with the activations and results of previous findings, mainly dealing with the upper limbs. In a motor system, lateral inhibition can result in the selection of one movement pattern with the suppression of others in the interests of specificity of movement. In upper-limb activity, it is common to observe a significant deactivation (i.e., decreased blood flow) in the ipsilateral sensorimotor cortex and subcortical regions and, when present, the contralateral cerebellum. Conjunction analysis demonstrated regions that are activated by one hand and deactivated by the contralateral hand [25]. However, this behavior has not yet been explored for the lower limb.

### 4.1. Implications for Practice

Lower-limb activity generates specific brain activity, confirming that motor control mechanisms differ between the upper and lower limbs. From the findings with healthy subjects, (re)learning strategies, specifically physiotherapy, need to promote the specific mechanisms for the movement control: the bilateral brain activation and the bilateral interconnectivity and function of the lower limbs, indicating the need for a bilateral approach to lower-limb movements and tasks coordination movement with contralateral stabilization. Despite the harmful impact of excessive activation of the unaffected hemisphere on stroke patients [26], the bilateral brain activation is important for normal brain behavior. Eventually, control of symmetric levels of activity of lower limbs is required to not stimulate the overuse of the unaffected limb and consequently of the unaffected hemisphere.

The type of stimulus also seems to be relevant when designing an intervention plan. Manual stimuli elicit cortical and subcortical brain activity in healthy subjects, while verbal stimuli only elicit cortical activation, implying that when we need to stimulate the subcortical areas, then manual stimulus without any verbal support might be appropriate. However, when looking for more cognitive stimuli, verbal or mixed stimuli would be more suitable. The presence of cingulate areas shows the importance of meaningful tasks for motor control in order to stimulate motivation and willingness for movement. These findings are important to validate the impact of manual therapeutic strategies and to develop physiological understanding for patients with neurological disorders. However, this needs further validation.

### 4.2. Research Implications

Considering the limited research of lower-limb and brain activity, our results can contribute to future development. However, maps alone are not sufficient for an understanding of cerebral processes. Remapping is neuronal and functionally driven; however, the proficiency of functional output can be constrained, if the map user does not use the newly remapped area correctly [27] applied to repeated meaningful tasks. Thus, specific regions of interest and connectivity studies are required to understand the mechanisms of motor control. The fine structure of the motor map appears not to be map-like at all, meaning that recovery processes within small areas may not be best interpreted as remapping. In fact, the characterization of changes in activity and connectivity that appear to support recovery as “reorganization” or “remapping” often seems overblown in situations in which synaptic strength and the excitability of preexisting circuits are adjusted [27]. Thus, the brain analysis of patients with neurological disorders is also of great importance in different phases of recovery.

With regard to the methods used in this study, we recommend fMRI procedures for functional sequences in the same run to minimize instrumental bias and to allow for direct comparisons between right and left limbs and to strengthen the validity of the results.

## 5. Conclusions

With regards to the goals of our study, we conclude that the brain somatotopic map for lower-limb multijoint movement is in line with previous findings on bilateral brain activation and the activation of cortical and subcortical areas. Furthermore, the activation of white matter is an important feature. Concerning the effects of the physiotherapeutic manual facilitation of lower-limb functional movements, we conclude that for healthy subjects manual facilitation promotes brain activity and that the areas activated are the same as those described above.

## Supplementary Material

Appendix 1. Presents the specific values of activation of the individual areas activated on each cluster, regarding the contrasts.

## Figures and Tables

**Table 1:** Subjects characteristics.

Subjects	Age	Gender	STAI Y1	SLUMS	STQ	Lateralization
1	84	F	34	25	23	Right
2	57	M	28	26	24	Right
3	60	M	32	30	14	Right
4	63	F	26	28	18	Right
5	56	F	28	25	19	Right
6	55	M	25	30	9	Right
7	52	F	43	25	15	Right
8	64	F	34	27	14	Right
9	56	M	25	30	17	Right
10	56	M	41	30	20	Right

Average	60,6	—	31,6	27,6	17,3	—

STAI Y1: State-Trait Anxiety Inventory (min. 20; max. 80); STQ: Social Touch Questionnaire (min. 0; max. 80); SLUMS: Saint Louis University Mental Status (min. 1; max. 30).

**Table 2:** Experimental paradigm.

	322 seconds, approx. 5 min per RUN				
	Fixation block Baseline 1	Block 1 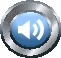	Block 2 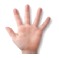	Block 3 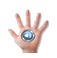	Fixation block Baseline 2
RUN 1, right lower-limb movement	30 seconds	Pseudorandomized sequence, with 5 repetitions of each block and 15 seconds of rest for replacing the lower limb to the initial position, in between each repetition			30 seconds

RUN 2, left lower-limb movement	30 seconds	Pseudorandomized sequence, with 5 repetitions of each block and 15 seconds of rest for replacing the lower limb to the initial position, in between each repetition			30 seconds

**(a):** 

Contrast	Run	Cluster	Center of Mass^*^			Region area	BA	Peak Voxel^*^			Region area	BA	Other BA included in the cluster	Number of voxels	*t*-test	*P* value	Function
			*x*	*y*	*z*			*x*	*y*	*z*							
Verbal versus baseline	Right	1	56,72	−16,26	6,09	Temporal lobe; superior temporal gyrus	42 R	47	−14	6	Temporal lobe; superior temporal gyrus	42 R	—	4425	6,30	0,000	Processing auditory information
		2	−2,3	−50,54	50,26	Parietal lobe; precuneus	S2-7 R	−1	−80	46	Parietal lobe; precuneus	S2-7 R	1, 2, 3, 4, 24 R	16655	6,59	0,000	Processing somatosensory and motor information (motivation and execution)
		3	−0,34	−55,52	3,8	Lingual gyrus	NA	−4	−62	3	Lingual gyrus	NA	—	3480	5,41	0,000	Visual recognition of words
		4	−34,97	−59,22	47,28	Parietal lobe; inferior parietal lobule	S2-7 L	−28	−65	55	Parietal lobe, superior parietal lobule	S2-7 L	1, 2, 3, 4, 6, 24 L	2080	5,60	0,000	Processing somatosensory and motor information (motivation, planning, and execution)
		5	−59,17	−21,42	6,55	Temporal lobe; superior temporal gyrus	42 L	−58	1	9	Temporal lobe; superior temporal gyrus	42 L	—	5177	6,10	0,000	Processing auditory information
	Left	1	51,8	−17,84	7,86	Temporal lobe; superior temporal gyrus	42 R	47	−20	6	Temporal lobe; superior temporal gyrus	42 R	—	3541	5,63	0,000	Processing auditory information
		2	1,71	−31,33	54,31	Frontal lobe, precentral gyrus	M1-4 R/L	−1	−32	60	Frontal lobe, precentral gyrus	M1-4 R/L	1, 2, 3, 5, 24 (R/L), 6 (L)	7153	5,03	0,000	Processing somatosensory and motor information (motivation, planning, and execution)
		3	−57,16	−13,99	5,48	Temporal lobe; superior temporal gyrus	42 L	−49	−23	9	Temporal lobe; superior temporal gyrus	42 L	—	3830	5,25	0,000	Processing auditory information

Manual versus baseline	Right	1	−5,04	−36,5	58,66	Parietal lobe; central gyrus	S1-1L	−4	−41	57	Parietal lobe, central gyrus	S1-1L	2, 3, 4, 5 L	2784	4,99	0,000	Processing somatosensory and motor information (execution)
		2	−5,06	−75,24	43,24	Parietal lobe; precuneus	S2-7L	−10	−71	48	Parietal lobe; precuneus	S2-7L	—	1064	4,48	0,000	Processing visuomotor coordination information
	Left	1	9,16	−37,59	55,61	Ventral cingulate cortex	24 R	38	−41	51	Superior parietal lobe	S2-5L	1, 2, 3, 4, 24 (R/L)	11004	5,30	0,000	Processing somatosensory and motor information (motivation, planning, and execution)

Verbal versus manual	Right	1	56,55	−17,03	6,34	Temporal lobe; primary auditory cortex	42 R	47	−14	6	Temporal lobe; primary auditory cortex	42 R	—	4802	6,30	0,000	Processing auditory information
		2	18,82	−67,3	26,33	Parietal lobe; precuneus	31 R	14	−62	15	Limbic lobe	31 R	—	1308	4,18	0,000	Processing emotions and recognition
		3	−0,84	−59,71	31,8	Parietal lobe; precuneus	S2-7 L	2	−44	39	Limbic lobe; cingulate gyrus	31 R	1, 5, 7 L	17222	6,67	0,000	Processing somatosensory information and emotions
		4	−23,82	−76,38	25,78	Occipital lobe	19 L	−19	−89	28	Occipital lobe	19 L	—	1429	3,89	0,000	Processing visual information
		5	−38,57	−48,11	46,84	Parietal lobe, inferior parietal lobule	40 L	−28	−65	54	Superior parietal lobe	S2-7 L	—	5018	5,85	0,000	Processing somatosensory information
		6	−60,01	−25,59	6,95	Temporal lobe	22 L	−61	−14	6	Temporal lobe	22 L	21	4892	6,12	0,000	Language comprehension
		7	−59,29	−0,49	−2,26	Temporal lobe	22 L	−62	4	0	Temporal lobe	22 L	21	1205	5,82	0,000	Language comprehension
	Left	1	54,79	−16,53	6,88	Temporal lobe; superior temporal gyrus	42 R	50	−8	3	Temporal lobe	22 R	—	3243	4,86	0,000	Processing auditory information and language comprehension
		2	−59,34	−11,55	4,06	Temporal lobe; superior temporal gyrus	22 L	−64	−20	−6	Temporal lobe; superior temporal gyrus	22 L	—	3350	5,35	0,000	Language comprehension

Manual versus verbal	Right	1	39,08	−64,17	12,73	Occipital lobe; middle occipital gyrus	19 R	35	−56	3	White matter; occipital lobe	NA	—	2701	4,38	0,000	Processing visual information
		2	9,92	30,9	−1,26	White matter; frontal lobe; prefrontal cortex R	NA	17	31	−3	White matter; frontal lobe; prefrontal cortex R	NA	—	1037	4,27	0,000	Executive functions
		3	−40,24	−61,92	9,41	Occipital lobe	19 L	−46	−59	6	Occipital lobe	19 L	—	958	5,04	0,000	Processing visual information
	Left	1	28,71	−22,78	30,62	Parietal lobe; subcentral gyrus; white matter R	NA	48	−35	51	Parietal lobe; subpostcentral gyrus; white matter R	NA	—	42752	5,44	0,000	Processing somatosensory information; connectivity with M1
		2	44,94	−46,16	−4,1	Temporal lobe; lateral occipitotemporal gyrus	37 R	44	−38	−3	Temporal lobe; lateral occipitotemporal gyrus	37 R	—	1604	4,41	0,000	Processing multimodal information
		3	34,57	−70,21	−0,07	Occipital lobe	19 R	38	−50	6	Occipital lobe	19 R	—	2835	4,89	0,000	Processing visual information
		4	21,29	−5,92	−8,72	Limbic lobe; amygdala R	NA	26	−2	−15	Limbic lobe; amygdala R	NA	—	1389	4,07	0,000	Processing emotional and motivational information
		5	15,83	−48,26	−35,17	Cerebellum posterior; lobes VIIIb and IX R	NA	14	−53	−33	Cerebellum posterior; lobes VIIIb and IX R	NA	—	1164	5,26	0,000	Processing somatosensory information
		6	−6,06	−29,09	−22,44	Brainstem; superior dorsal pons L	NA	−1	−29	−24	Brainstem; superior dorsal pons L	NA	—	1789	4,78	0,000	Communication with the cerebellum
		7	−15,6	−20,15	5,25	Thalamus; ventroposterolateral nucleus L	NA	−7	−14	9	Thalamus; ventroposterolateral nucleus L	NA	—	2291	4,91	0,000	Processing somatosensory information
		8	−25,32	−19,4	32,93	Parietal lobe; subcentral gyrus; white matter L	NA	−25	−20	30	Parietal lobe, subcentral gyrus; white matter L	NA	—	13258	4,76	0,000	Processing somatosensory information; connectivity with M1
		9	−20,93	−41	−33	Cerebellum posterior; lobes VIIIb and IX R	NA	−19	−38	−27	Cerebellum posterior; lobes VIIIb and IX R	NA	—	1485	5,13	0,000	Processing somatosensory information
		10	−33,53	4,33	−7,23	Insula lobe L	NA	−34	−5	−3	Insula lobe L	NA	—	1601	3,55	0,001	Processing emotions
		11	−47,05	−13,71	−13,92	Temporal lobe; subgyral L	21	−49	−29	−9	Temporal lobe; subgyral L	21	—	1521	5,46	0,000	Processing auditory and language information

Manual + verbal versus baseline	Right	1	53,52	−17,26	7,23	Temporal lobe; superior temporal gyrus	42 R	59	−17	0	Temporal lobe; superior temporal gyrus	22 R	—	5054	5,50	0,000	Processing auditory information and language comprehension
		2	−3,88	−37,85	59,07	Parietal lobe, postcentral gyrus	1 L	−4	−41	57	Parietal lobe, postcentral gyrus	2 L	—	2343	5,00	0,000	Processing somatosensory information
		3	57,75	−23,57	7,35	Temporal lobe; superior temporal gyrus	42 L	−64	−32	6	Temporal lobe; superior temporal gyrus	22 L	—	4276	6,02	0,000	Processing auditory information and language comprehension
	Left	1	50,65	−20,25	9,91	Temporal lobe; superior temporal gyrus	42 R	50	5	3	Temporal lobe; superior temporal gyrus	22 R	—	9426	6,61	0,000	Processing auditory information and language comprehension
		2	4,01	−32,12	54,61	Frontal lobe; cingulate cortex ventral	24 R	20	−35	57	Parietal lobe; prepyriform cortex	5 R	—	6161	4,61	0,000	Processing somatosensory and motivation information
		3	−55,53	−19,25	7,6	Temporal lobe	22 L	−52	−17	6	Temporal lobe	22 L	—	4829	5,60	0,000	Language comprehension

^*^Talairach coordinates; BA: Brodmann area; R: right hemisphere; L: left hemisphere; S2: secondary somatosensory area; S1: primary somatosensory area; M1: primary motor area.

**(b):** 

Contrast	Run	Cluster	Center of Mass^*^			Region area	BA	Peak Voxel^*^			Region area	BA	Number of voxels	*t*-test	*P* value	Function
			*x*	*y*	*z*			*x*	*y*	*z*						
Verbal versus baseline	Right	1	0,36	−6,54	6,8	Ventral interhemispheric region; commissures	NA	44	−8	−18	Temporal lobe; inferior temporal gyrus	20 R	246561	−6,96	0,000	Processing interhemispheric connectivity; visual object recognition
	Left	1	−3,43	−31,28	0,21	Parahippocampal gyrus	27 L	29	−86	15	Occipital lobe; middle occipital gyrus	18 L	307282	−8,60	0,000	Processing memory and visual information

		1	27,76	−6,41	25,44	Frontal lobe; subprecentral gyrus; white matter R	NA	50	−8	30	Frontal lobe; precentral gyrus	4 R	13466	−5,39	0,000	Processing motor information
		2	33,56	−17,33	−14,22	Limbic lobe; parahippocampal gyrus; white matter R	NA	26	−20	−15	Limbic lobe; parahippocampal gyrus; white matter R	NA	2347	−5,57	0,000	Processing complex aspects of learning and memory
		3	29,23	−79,82	14,5	Occipital lobe	18 R	29	−71	21	Occipital lobe	19 R	2161	−4,21	0,000	Processing visual information
Manual versus baseline	Right	4	−22,84	16,07	22,1	Frontal lobe; subsuperior frontal gyrus white matter L	NA	−25	43	30	Frontal lobe; superior frontal gyrus	9 L	29285	−6,16	0,000	Processing executive information
		5	7,37	45,45	40,41	Frontal lobe; medial frontal gyrus	9 R	5	52	36	Frontal lobe; medial frontal gyrus	9 R	1057	−4,09	0,000	Processing executive information
		6	−17,48	−44,53	−21,06	Cerebellum anterior; lobe V L	NA	−16	−41	−21	Cerebellum anterior; lobe V L	NA	3574	−5,20	0,000	Processing upper-limb motor information
		7	−25,33	−87,28	5,79	Occipital lobe, middle occipital gyrus	17 L	−28	−86	6	Occipital lobe, middle occipital gyrus	17 L	2314	−4,02	0,000	Processing visual information
		8	−36,44	−1,44	−29,22	Temporal lobe; inferior temporal gyrus; white matter L	NA	−43	−11	−21	Temporal lobe; inferior temporal gyrus; white matter L	White matter L-temporal	2547	−4,37	0,000	Processing auditory and language information
		9	−46,5	−35,55	−6,36	Temporal lobe; inferior temporal gyrus; white matter L	NA	−25	−44	−6	Temporal lobe; inferior temporal gyrus; white matter L	37 L	1103	−4,16	0,000	Multimodal integration, faces and object recognition
		10	−41,74	8,28	−18,05	Temporal lobe; inferior temporal gyrus; white matter L	37 L	−49	7	−12	Temporal lobe; inferior temporal gyrus; white matter L	37 L	2016	−4,49	0,000	Multimodal integration, faces and object recognition
		11	−43	−51,96	−36,83	Cerebellum posterior; lobe crus I L	NA	−40	−44	−30	Cerebellum posterior; lobe crus I L	NA	1732	−5,49	0,000	Processing language and memory information
	Left	1	2,52	−70,52	−13,26	Cerebellum posterior; lobe VI proximal R	NA	−7	−98	3	Occipital lobe, middle occipital gyrus	17 R	34619	−5,36	0,000	Processing upper-limb movement and visual information
		2	33,08	3,74	−28,17	Temporal lobe; medial temporal gyrus	38 R	32	19	−33	Temporal lobe; medial temporal gyrus	38 R	5782	−6,05	0,000	Processing emotional and memory information
		3	4,67	49,87	38,23	Frontal lobe	9 R	20	49	36	Frontal lobe	9 R	2230	−4,70	0,000	Processing cognitive and execution information
		4	−13,68	43,68	5,96	Frontal lobe; subsuperior frontal gyrus white matter L	NA	−16	37	6	Frontal lobe; subsuperior frontal gyrus white matter L	NA	5707	−4,73	0,000	Processing executive information
		5	−39,05	44,1	18,74	Frontal lobe	9 L	−40	43	21	Frontal lobe	9 L	1367	−4,24	0,000	Processing cognitive and execution information

Verbal versus manual	Right	1	41,3	−2,06	−23,03	Temporal lobe; inferior temporal gyrus; white matter R	NA	44	−8	−18	Temporal lobe; inferior temporal gyrus; white matter R	NA	5003	−5,89	0,000	Processing auditory and language information
		2	39,65	−57,88	−5,58	Occipital lobe, inferior occipital gyrus R	NA	44	−59	−3	Occipital lobe, inferior occipital gyrus R	NA	3409	−4,70	0,000	Processing visual information
		3	30,17	−27,48	−7,28	Limbic lobe; hippocampus gray matter R	NA	32	−26	−9	Limbic lobe; hippocampus gray matter R	NA	1053	−5,57	0,000	Processing memory information
		4	26,52	3,62	−10,78	Insula lobe R	NA	32	13	−6	Insula lobe R	NA	1369	−4,57	0,000	Processing auditory somesthetic skeletomotor function
		5	27,49	−8,38	20,28	Frontal lobe; subprecentral gyrus white matter R	NA	23	−14	27	Frontal lobe; subprecentral gyrus white matter R	NA	1260	−4,01	0,000	Processing motor information
		6	8,85	33,23	14,28	Frontal lobe; cingulate gyrus; white matter R	NA	14	19	9	Subcortical area; caudate nuclei R	NA	8392	−4,57	0,000	Processing motor information (planning)
		7	−33,91	5,07	−7,05	Insula lobe L	NA	−40	7	−6	Temporal lobe; superior temporal gyrus; white matter L	NA	1260	−5,95	0,000	Processing auditory somesthetic skeletomotor function
		8	−33,58	−0,63	−30,26	Temporal lobe; inferior temporal gyrus; white matter L	White matter L-temporal	−28	4	−30	Temporal lobe; inferior temporal gyrus; white matter L	NA	2632	−5,14	0,000	Multimodal integration, faces and object recognition
	Left	1	0,79	−42,06	−5,38	Cerebellum anterior; lobe III R	NA	−7	−20	−21	Pons L	NA	57558	−6,53	0,000	Processing upper-limb function
		2	29,44	−5,55	25,09	Frontal lobe; subprecentral gyrus white matter R	NA	26	−5	33	Frontal lobe; subprecentral gyrus white matter R	NA	2564	−4,60	0,000	Processing motor information
		3	14,41	−45,97	21,39	Occipital lobe R	18 R	14	−59	27	Occipital lobe, precuneus R	31 R	1717	−4,58	0,000	Processing visual information
		4	−15,2	57,39	22,01	Frontal lobe; subsuperior frontal gyrus white matter L	NA	−16	61	21	Frontal lobe; subsuperior frontal gyrus white matter L	NA	1103	−4,03	0,000	Processing motor information
		5	−21,81	−66,95	−36,32	Cerebellum posterior; lobe crus II/VIIb L	NA	−7	−65	−39	Cerebellum posterior; lobe VIIIb L	NA	3873	−4,73	0,000	Processing somatosensory information
		6	−25,89	−88,88	3,86	Occipital lobe, middle occipital gyrus	17 L	−37	−90	6	Occipital lobe, middle occipital gyrus	19 L	1834	−5,14	0,000	Processing visual information
		7	−41,05	−45,4	−31,01	Cerebellum anterior lobe crus I L	NA	−37	−44	−30	Cerebellum anterior lobe crus I L	NA	1130	−5,41	0,000	Processing emotions

Manual versus verbal	Right	1	58,72	−15,02	6,52	Temporal lobe; superior temporal gyrus	42 R	56	−11	3	Temporal lobe; superior temporal gyrus	42 R	3128	−4,97	0,000	Processing auditory information
		2	−44,06	−24,24	45,7	Parietal lobe; precuneus	S2-7 L	−52	−17	39	Parietal lobe; precuneus	S2-7 L	1875	−4,52	0,000	Processing somatosensory information
		3	−59,67	−24,72	7,95	Temporal lobe	22 L	−61	−14	6	Temporal lobe	22 L	1485	−5,38	0,000	Language comprehension
	Left	1	59,51	−16,47	4,16	Temporal lobe; superior temporal gyrus	42 R	69	−23	0	Temporal lobe; superior temporal gyrus	42 R	2132	−4,76	0,000	Processing auditory information

Manual + verbal versus baseline	Right	1	−0,67	−6,14	7,7	Subcortical; thalamus L	NA	−25	−8	9	Subcortical; putamen L	NA	160427	−6,25	0,000	Processing motor information (planning)
		2	37,06	−61,71	−35,41	Cerebellum anterior lobe crus I R	NA	41	−47	−36	Cerebellum anterior lobe crus I R	NA	2654	−4,76	0,000	Processing emotions
		3	−39,41	−51,59	−34,95	Cerebellum anterior lobe crus I L	NA	−46	−44	−43	Cerebellum anterior lobe crus I L	NA	2849	−4,45	0,000	Processing emotions
	Left	1	−3,87	−54,99	−10,82	Cerebellum anterior; lobe V L	NA	−40	−47	36	Parietal lobe; supramarginal gyrus; white matter L	NA	125170	−6,94	0,000	Processing somatosensory information
		2	18,02	−42,09	30,2	Parietal lobe; subparietal gyrus; white matter R	NA	20	−26	24	Parietal lobe; subparietal gyrus; white matter R	NA	3346	−5,02	0,000	Processing somatosensory information
		3	−20,56	35,47	17,79	Frontal lobe; sub- middle frontal gyrus L	NA	−16	31	6	Frontal lobe; sub-middle frontal gyrus L	NA	14126	−6,83	0,000	Processing motor information (planning)

^*^Talairach coordinates; BA: Brodmann area; R: right hemisphere; L: left hemisphere; S2: secondary somatosensory area.
