# Supplementary material for: Brain Activity during Lower-Limb Movement with Manual Facilitation: An fMRI Study
Source: Neurol Res Int. 2015 Feb 2;2015:701452. doi: 10.1155/2015/701452 (PMC4333285; doi:10.1155/2015/701452)
Supplement: Supplementary file 1 — Appendix 1. Presents the specific values of activation of the individual areas activated on each cluster, regarding the contrasts. [file 701452.f1.pdf]

Appendix 1 - Brain areas of activation and Talairach coordinates

Right Stimulation - Control Group			
**Contrasts**	**B.A**	**Peak Voxel**	**t***
Verbal vs Baseline	**BA1, BA2, BA3 -** *Somatosensorial Homunculus* (right, left)	2, -35, 57<br>-4, -41, 57	4,576<br>5,018
	**BA4 -** *Primary motor cortex* (right, left)	1, -35, 60<br>-1, -35, 60	4,400<br>4,506
	**BA6 -** *Premotor cortex* (left)	-4, -5, 51	4,761
	**BA7** and **BA5** - *Secondary Somatosensorial Cortex* (right, left)	2, -47, 48<br>-28, -65, 55	5,077<br>5,604
	**BA18 -** *Extraestriate cortex V2* (right, left)	2, -61, 3<br>-4, -59, 4	4,995<br>4,580
	**BA21** - *Lateral temporal lobe* (right, left)	59, -19, 0<br>-61, -32, 3	3,494<br>4,737
	**BA22**- *Posterior parte contains Wernicke´s area* (right, left)	59, -5, 3<br>-61, -14, 6	5,347<br>4,801
	**BA24 -** *Cingulate cortex (ventral)* (right, left)	1, -38, 51<br>-2, -38, 51	3,597<br>4,238
	**BA30-** *Retroesplenial Agranular córtex/ Cingulate gyrus* (right, left)	5, -47, 3<br>-4, -53, 6	4,600<br>4,169
	**BA31** - *Isthmus of Cingulate gyrus* (right, left)	2, -59, 36<br>-1, -47, 48	3,729<br>4,552
	**BA38 -** *Temporal Pole* (left)	-61, 4, 0	4,868
	**BA42 -** *A1* (right, left)	59, -15, 12<br>-58, -32, 12	4,605<br>4,748
	Lingual Gyrus **(right, left)**	2, -61, 3	4,995

		-4, -59, 3	5,231
Manual vs Baseline	**BA1, BA2, BA3 -** *Somatosensorial Homunculus* (left)	-4, -41, 57	4,990
	**BA4 -** *Primary motor cortex* (left)	-15, -29, 63	3,783
	**BA5 and BA7-** *Secondary somatosensorial cortex* (left)	-10, -71, 48	4,477
Verbal+Manual vs Baseline	**BA1+3 -** *Somatosensorial Homunculus* (left)	-4, -41, 57	5,003
	**BA4 -** *Primary motor cortex* (left)	-1, -23, 63	3,497
	**BA5** - *Secondary Somatosensorial Cortex* (left)	-4, -41, 57	5,003
	**BA21** - *Lateral temporal lobe* (right, left)	59, -19,0<br>-61, -29, 3	4,425<br>4,271
	**BA22**- *Posterior parte contains Wernicke´s area* (right, left)	59, -17, 0<br>-63, -32, 6	5,502<br>5,407
	**BA24 -** *Cingulate cortex (Ventral)* (left)	-4, -41, 55	3,388
	**BA41 and BA42 -** *A1* (right, left)	62, -17, 6<br>-58, -32, 12	4,831<br>5,015
Verbal vs Manual	**BA1 -** *Somatosensorial Homunculus* (left)	-38, -23, 45	3,364
	**BA7** and **BA5** - *Secondary Somatosensorial Cortex* (left)	-1, -51, 45	4,701
	**BA18 and BA 19 -** *Extraestriate cortex V2* (left)	-4, -58, 3	4,229
	**BA21** - *Lateral temporal lobe* (right, left)	64, -23, 6<br>-61, -32, 3	5,261<br>4,749
	**BA22**- *Posterior parte contains Wernicke´s area* (right, left)	47, -14, 6<br>-61, -14, 6	6,297<br>6,116
	**BA24** - *Cingulate cortex (ventral)* (left)	-1, -36, 50	3,454
	**BA30-** *Retroesplenial Agranular cortex* (right, left)	5, -47, 3<br>-4, -50, 9	4,385<br>3,908
	**BA31** - *Isthmus of Cingulate gyrus* (right, left)	14, -62, 15<br>-4, -50, 39	4,178<br>4,814
	**BA38 -** *Temporal Pole* (left)	-61, 4, 0	5,824
	**BA41 and BA42 -** *A1* (right), **BA 42 -** A1 (left)	59, -15, 12<br>-58, -32, 12	4,524<br>4,218

	Lingual Gyrus (right, left)	5, -62, -3<br>-4, -59, 3	4,600<br>4,997
Manual vs Verbal	**BA18 + BA19 -** *Extraestriate cortex V2* (right, left)	44, -56, -3<br>-46, -59, 6	4,230<br>5,044
	Orbital surface (right, left)	17, 31, -3<br>-1, 28, -3	4,274<br>3,203
Verbal vs Manual + Verbal	**BA3 -** *Somatosensorial Homunculus* (right), **BA1, 2** and **3** (left)	1, -36, 60<br>-4,-44, 57	5,640<br>3,767
	**BA7 and BA5** - *Secondary Somatosensorial Cortex* (right, left)	20,-65, 53<br>-34, -59, 48	4,666<br>5,267
	**BA18 and BA 19 -** *Extraestriate cortex V2* (right, left)	14, -56, 18<br>-25, -80, 30	4,180<br>3,538
	**BA24** - *Cingulate cortex (ventral)* (right, left)	1, -35, 51<br>-1, -37, 50	2,517<br>2,905
	**BA30-** *Retroesplenial Agranular cortex* (right, left)	14, -53, 9<br>-4, -53, 6	4,441<br>4,441
	**BA31** - *Isthmus of Cingulate gyrus* (right, left)	2, -44, 39<br>-4, -53, 35	4,045<br>3,990
Manual + Verbal vs Verbal			
Manual vs Manual + Verbal			
Manual + Verbal vs Manual	**BA7 and BA5** - *Secondary Somatosensorial Cortex* (right, left)	2, -59, 36<br>-1, -50, 45	4,139<br>3, 529
	**BA22 -** *Wernicke* - (right, left)	59, -17, 0<br>-61, -23, 9	5,291<br>4,385
	**BA41 and BA42 -** *A1* (right), **BA42** (right)	62, -17, 6<br>-58, -32, 12	4,599<br>4,436

Left Stimulation - Control Group			
**Contrasts**	**B.A**	**Peak Voxel**	**t***
Verbal vs Baseline	**BA1, BA2, BA3 -** *Somatosensorial Homunculus* (right, left)	2, -44, 57<br>-1, -50, 57	4,135<br>4,196
	**BA4 -** *Primary motor cortex* (right, left)	1, -33, 60<br>-1, -32, 60	4,480<br>5,026
	**BA5** - *Secondary somatosensorial cortex* (right, left)	20, -35, 60<br>-3, -53, 57	4,052<br>3,630
	**BA6 -** *Premotor cortex* (left)	-4, 4, 42	5,513
	**BA21** - *Lateral temporal lobe* (right, left)	50, -23, 6<br>-49, -23, 9	5,310<br>5,252
	**BA22**- *Posterior parte contains Wernicke´s area* (right, left)	50, -3, 3<br>-59, -8, 6	3.,722<br>3,691
	**BA24 -** *Cingulate cortex (Ventral part)* (right, left)	5, -38, 48<br>-4, -33, 48	3,663<br>4,498
	**BA38 -** *Temporal Pole* (left)	-58, 7, 0	4,600
	**BA41 and BA42 -** *A1* (right, left)	47, -18, 7<br>-55, -18, 9	3,879<br>4,525
Manual vs Baseline	**BA1, BA2, BA3 -** *Somatosensorial Homunculus* (right, left)	11, -41, 60<br>-7, -38, 51	4,408<br>3,837
	**BA4 -** *Primary motor cortex* (right, left)	8, -37, 66<br>-1, -32, 57	4,759<br>3,781
	**BA5 and BA7-** *Secondary somatosensorial cortex* (right)/ **BA5** (left)	38, -41, 51<br>-16, -51, 57	5,300<br>3,910
	**BA24** - *Cingulate Cortex* (ventral) (right, left)	5, -38, 48	4,119

		-4, -33, 48	3,508
	**BA32 -** *Anterior Cingulate* (right, left)	5, -17, 45<br>-1, -17, 51	3,781<br>3,070
Verbal+Manual vs Baseline	**BA1, BA2 and BA3 -** *Somatosensorial Homunculus* (right) / **BA3** (left)	11, -41, 60<br>-4, -35, 50	4,274<br>4,115
	**BA4 -** *Primary motor cortex* (right, left)	8, -37, 66<br>-1, -32, 60	3,633<br>4,139
	**BA5** - *Secondary Somatosensorial Cortex* (right, left)	20, -35, 57<br>-2, -50, 54	4,608<br>2,694
	**BA21** - *Lateral temporal lobe* (right, left)	62, -23, 0<br>-47, -26, 6	4,321<br>3,043
	**BA22**- *Posterior parte contains Wernicke´s area* (right, left)	50, -5, 4<br>-58, -7, 6	5,171<br>4,324
	**BA24 -** *Cingulate Cortex* (ventral) (right, left)	5, -38, 48<br>-4, -35, 49	4,394<br>4,328
	**BA38 -** *Temporal Pole* (left)	-58, 7, 0	4,327
	**BA41 and BA42 -** *A1* (right, left)	47, -18, 7<br>-55, -18, 9	4,975<br>4,388
Verbal vs Manual	**BA21** - *Lateral temporal lobe* (left)	-64, -20, -6	5,354
	**BA22**- *Posterior parte contains Wernicke´s area* (right, left)	50, -5, 6<br>-63, -11, 6	3,863<br>3,832
	**BA38** - *Temporal Pole* (left)	-58, 7, 0	3,716
	**BA42 -** *A1* (right, left)	50, -15, 9<br>-55, -17, 9	3,582<br>5,067
Manual vs Verbal	**BA1**, **BA2, BA3** - *S1* (right, left)	9, -35, 72<br>-16, -35, 51	4,655<br>3,875
	**BA4** - *M1* (right, left)	26, -13, 63	3,927

		-40, -11, 33	3,083
	**BA6 – Premotor** (right, left)	38, -2, 42<br>-40, -2, 42	5,112<br>3,192
	**BA5** and **BA7 -** *S2* (right, left)	48, -35, 51<br>16, -35, 51	5,441<br>3,878
	**BA10 -** *Prefrontal Cortex* (right)	11, 38, 15	3,918
	**BA17 -** *V1* (right)	17, -47, 6	3,221
	**BA18 and BA19 -** *V2* (right)	29, -89, -6	4,144
	**BA21 -** *Lateral Temporal Lobe* (left)	-52, -17, -12	3,732
	**BA23 -** *Cingulate Cortex - Posterior* (right, left)	2, -38, 24<br>-7, -44, 30	3, 457<br>3,716
	**BA24 -** *Cingulate Cortex - Ventral* (right, left)	11, -22, 36<br>-13, -32, 42	4,031<br>2,873
	**BA30 -** *Retroesplenial Agranular Cortex* (right, left)	2, -38, 20<br>-4, -41, 15	2,755<br>3,947
	**BA31-** *Isthmus - Cingulate córtex* (right, left)	5, -53, 15<br>-4, -35, 36	3,227<br>3,755
	**BA32 -** *Anterior Cingulate* (right)	8, -17, 44	3,141
	**BA37-** *Fusiform gyrus - caudal* (right)	38, -47, -6	4,398
	**BA39 -** *Angular gyrus* (right)	53, -41, 18	4,106
	**BA40 -** *Secondary Somatosensorial representation* (right)	53, -35, 33	4,187
	**BA44 -** *Broca* (right, left)	38, 13, 30<br>-37, 13, 21	3,840<br>4,177
	**BA46** - *Dorsolateral Prefrontal Cortex* - (right)	35, 31, 15	4,291
	Anterior Lobe Cerebellum (right, left)	17, -41, -30<br>-19, -38, -27	3,414<br>5,131
	Posterior Lobe Cerebellum (right)	14, -53, -33<br>-14, -53, -33	5,260

		(esq.)	
	Amygdala (right)	26, -2, -15	4,069
	Thalamus (right, left)	23, -29, 6<br>-7, -14, 9	4,864<br>4,905
	Brainstem-Pons (left)	-1, -29, -24	4,776
Verbal vs Manual + Verbal			
Manual + Verbal vs Verbal	**BA4 -** *M1* (right)	26, -11, 48	3,386
	**BA6** - *Premotor cortex* (right)	38, -2, 36	4,410
	**BA7 -** *S2* (right)	44, -41, 36	3,330
	**BA10 -** *Prefrontal cortex* (right)	5, 49, 27	3,651
	**BA18** and **BA19 -** *V2* (right)	35, -62, 0	3,882
	**BA21** - *Lateral Temporal Lobe* (right)	41, -20, -3	3,597
	**BA22 -** *Wernicke* (right)	47, -11, -9	3,718
	**BA32 -** *Anterior Cingulate* (right)	11, 37, 15	2,996
	**BA37 -** *Fusiform Gyrus* (right)	45, -40, 12	2,585
	**BA39 -** *Angular Gyrus* (right)	44, -38, 12	3,217
	**BA40** - *Secondary somatosensorial representation* (right)	53, -37, 33	3,215
	**BA41** and **BA42** - *A1* (right)	32, -23, 15	4,106
Manual vs Manual + Verbal	**BA5** and **BA7 -** *S2* (right)	26, -47, 42	3,396
	**BA31-** *Isthmus - Cingulate córtex* (right)	14, -35, 39	3,393
Manual + Verbal vs Manual	**BA21 -** *Lateral Temporal lobe* (right, left)	62, -23, 0<br>-64, -20, -6	4,587<br>3,304
	**BA22 -** *Wernicke* (right, left)	63, -20, 6<br>-59, -8, 6	4,325<br>3,794
	**BA38 -** *Temporal Pole* (left)	-58, 7,0	3,142
	**BA42 -** *A1* (right, left)	62, -18, 9<br>-55, -17, 9	4,073<br>5,064
